# Characterization of the pharmacokinetics of entrectinib and its active M5 metabolite in healthy volunteers and patients with solid tumors

**DOI:** 10.1007/s10637-020-01047-5

**Published:** 2021-01-18

**Authors:** Georgina Meneses-Lorente, Darren Bentley, Elena Guerini, Karey Kowalski, Edna Chow-Maneval, Li Yu, Andreas Brink, Nassim Djebli, Francois Mercier, Vincent Buchheit, Alex Phipps

**Affiliations:** 1 Roche Innovation Centre Welwyn, Roche Products Ltd, Welwyn Garden City, UK; 2 Certara Strategic Consulting, London, UK; 3 Roche Innovation Center Basel, F. Hoffmann-La Roche, Basel, Switzerland; 4 Ignyta, San Diego, CA USA; 5 Roche Innovation Center, Little Falls, NJ USA

**Keywords:** Entrectinib, TRK/ROS1/ALK, Pharmacokinetics, Bioequivalence, Food effect

## Abstract

*Background*: Entrectinib is an oral, CNS-active, potent inhibitor of tyrosine receptor kinases A/B/C, tyrosine kinase ROS proto-oncogene 1, and anaplastic lymphoma kinase approved for use in patients with solid tumors. We describe 3 clinical studies, including one investigating the single/multiple dose pharmacokinetics of entrectinib in patients and two studies in healthy volunteers investigating the absorption/distribution/metabolism/excretion (ADME) of entrectinib, its relative bioavailability, and effect of food on pharmacokinetics. *Methods*: The patient study is open-label with dose-escalation and expansion phases. Volunteers received entrectinib (100–400 mg/m^2^, and 600–800 mg) once daily with food in continuous 28-day cycles. In the ADME study, volunteers received a single oral dose of [^14^C]entrectinib 600 mg. In the third study, volunteers received single doses of entrectinib 600 mg as the research and marketed formulations in the fasted state (Part 1), and the marketed formulation in the fed and fasted states (Part 2). Entrectinib and its major active metabolite M5 were assessed in all studies. *Results*: Entrectinib was absorbed in a dose-dependent manner with maximum concentrations at ~4 h postdose and an elimination half-life of ~20 h. Entrectinib was cleared mainly through metabolism and both entrectinib and metabolites were eliminated mainly in feces (minimal renal excretion). At steady-state, the M5-to-entrectinib AUC ratio was 0.5 (with 600 mg entrectinib research formulation in patients). The research and marketed formulations were bioequivalent and food had no relevant effect on pharmacokinetics. *Conclusions*: Entrectinib is well absorbed, with linear PK that is suitable for once-daily dosing, and can be taken with or without food.

**Supplementary Information:**

The online version contains supplementary material available at 10.1007/s10637-020-01047-5.

## Introduction

Entrectinib (also known as RXDX-101 and Rozlytrek®) is a CNS-active, potent inhibitor of tyrosine receptor kinases (TRK) A, B and C, tyrosine kinase ROS proto-oncogene 1 (ROS1) and anaplastic lymphoma kinase (ALK). These kinases are overexpressed or dysregulated in cancer with constitutive activity, making the growth of the cancer cells dependent on the abnormal kinases [1, 2]. Molecular alterations in kinases are found in many types of cancer and therefore represent attractive targets for anticancer therapy [3]. Entrectinib has been shown to have antitumor activity in advanced and/or metastatic solid tumors [4–6]. Entrectinib received its first global approval in Japan for the treatment of adult and pediatric patients with neurotrophic TRK (NTRK) fusion-positive, advanced or recurrent solid tumors. The U.S. Food and Drug Administration (FDA) has since approved entrectinib for the treatment of adults with ROS1 fusion-positive, metastatic non-small cell lung cancer (NSCLC) and granted accelerated approval for the treatment of adult and pediatric patients 12 years of age and older with NTRK fusion-positive solid tumors [7]. The recommended dosage for adults with ROS1 fusion-positive NSCLC or NTRK fusion-positive solid tumors is 600 mg once daily orally and was determined using a maximum tolerated dose (MTD) approach from the dose escalation study STARTRK-01 [4–6].

Entrectinib is a lipophilic, basic, moderately permeable molecule with strongly pH-dependent solubility [8]. A total of three different clinical capsule formulations (F1, F2A, and F06) have been used in the patient clinical studies. Based on exploratory observations from Study ALKA-372-001 [5, 6], performance of the F1 formulation appeared to be sub-optimal during clinical dosing due to the formulation performance being very sensitive to conditions in the gastro-environmental tract, which is consistent with recently reported GastroPlus data [8]. Subsequently, an alternative gelatin capsule formulation (F2A) was developed. This formulation contained an acidulant (betaine hydrochloride) in order to reduce the sensitivity and variability of entrectinib bioavailability to gastric pH conditions. Further formulation development resulted in the marketed formulation F06 which also included an acidulant (tartaric acid) as a functional excipient [8]. The preclinical pharmacokinetics (PK) of entrectinib have been evaluated in mice, rats and dogs to support the preclinical safety and PK assessments of entrectinib [7]. Entrectinib is characterized by low total plasma clearance, a large volume of distribution, high plasma protein binding, and a moderately long terminal half-life (3.5–11.9 h) in all species. Single oral-dose administration of entrectinib in a solution formulation showed moderate-to-high absolute bioavailability (31–76%) compared with intravenous (IV) administration in preclinical studies. In vitro metabolism studies showed that entrectinib is metabolized by cytochrome P450 3A (CYP3A) to its major similarly active metabolite, M5 [7]. In vivo, entrectinib and its metabolites were excreted mainly in the feces (>97%%) in rats following either an IV or oral dose.

Here we report pharmacokinetic (PK) results from 3 clinical studies in adults (1 in patients and 2 in healthy volunteers) which were designed to investigate the single and multiple dose pharmacokinetics of entrectinib and its major active metabolite (M5; the desmethyl metabolite) after oral entrectinib administration. The studies also investigated entrectinib disposition, and the effect of different capsule formulations and food on the PK of entrectinib.

## Methods

### Study design

Study 1 (STARTRK-1; NCT02097810) is an open-label study with dose-escalation and dose expansion phases in adult patients with advanced/metastatic solid tumors. The dose escalation phase is complete and is discussed here. The dose expansion phase is still ongoing and is not discussed. An objective of the study was to investigate the single and multiple-dose PK of entrectinib and M5 over a range of doses. Patients initially received entrectinib doses based on body surface area (100, 200 and 400 mg/m^2^/day), and subsequently as flat doses (600 or 800 mg/day). All doses were administered once daily under fed conditions (within 1 h after a meal) in continuous 28-day cycles. Two entrectinib capsule formulations were investigated; F1 which included standard, non-functional excipients, and F2A which included an acidulant. All entrectinib dose levels were investigated using the F1 formulation. In addition, the 600 mg flat dose was also investigated using the F2A formulation. Blood samples for entrectinib and M5 plasma concentrations were collected at intervals, including 24-h profiles on Days 1, 14, and 28 of Cycle 1.

Study 2 was an open-label study designed to assess the absorption, distribution, metabolism and excretion (ADME) of a single dose of [^14^C]entrectinib in healthy adult volunteers. Volunteers received 600 mg (~200 μCi) of [^14^C]entrectinib (with an acidulant to mimic the F2A formulation) after a 10- h fast. Blood, urine, and fecal samples were collected up to 312 h postdose to measure total radioactivity (plasma, whole blood, urine and feces), and entrectinib and M5 concentrations (plasma and urine). Metabolic profiles were also evaluated in plasma, urine, and feces.

Study 3 was a 2-part study in healthy adult volunteers designed to investigate the bioequivalence of the research capsules (F2A) of entrectinib used in the pivotal clinical studies and the marketed capsules (F06, which also contained an acidulant) under fasted conditions, and also to investigate the effect of food on the PK of entrectinib administered as the marketed capsules. Both parts were open-label, randomized, 2-way crossover designs. In Part 1, the volunteers received a single oral dose of 600 mg entrectinib under fasted conditions as F2A capsules and F06 capsules in random order. In Part 2, the volunteers received a single oral dose of 600 mg entrectinib as F06 capsules under either fed (high-fat, high-calorie meal) or fasted conditions in random order. The timing and content of the high-fat, high-calorie breakfast was in accordance with the US Food and Drug Administration guideline for food-effect studies [9]. In both parts, the 2 treatments were separated by a 9-day washout period. Blood samples were collected up to 120 h postdose to measure plasma entrectinib and M5 concentrations.

### Participants

Study 1 included adult male and female patients who had a histologically or cytologically confirmed diagnosis of relapsed or refractory locally advanced or metastatic solid tumors for whom no alternative effective standard therapy was available or for whom standard therapy was considered unsuitable or intolerable. In the dose escalation phase, it was preferred, but not required to, enroll patients with tumors harboring NTRK1, NTRK2, NTRK3, ROS1, or ALK molecular alterations. Patients had measurable or evaluable disease using Response Evaluation Criteria in Solid Tumors (RECIST) v1.1 and a life expectancy of at least 3 months. Prior cancer therapy was allowed, but had to have been completed within prespecified time-limits prior to the start of entrectinib dosing. Use of moderate cytochrome (CYP) 3A inducers was allowed at the discretion of the investigator but had to have been stable or decreasing in dose for at least 2 weeks prior to the start of entrectinib dosing.

Studies 2 and 3 included healthy male adult volunteers. Key exclusion criteria included restrictions of any prescription and non-prescription drugs, herbal remedies, or vitamin supplements for at least 14 days prior to, and throughout the studies. Specifically, no drugs known to be significant inhibitors or inducers of CYP enzymes, P-glycoprotein, organic anion-transporting polypeptide, or acid-reducing agents were allowed.

All patients and healthy volunteers had to provide written informed consent prior to study participation.

### Pharmacokinetic assessments

In all 3 studies, entrectinib and M5 plasma concentrations were measured using a validated liquid chromatography-tandem mass spectrometry (LC-MS/MS) method with a lower limit of quantification of 2.00 ng/mL. Radioactivity was determined using liquid scintillation counting. PK parameters were determined using noncompartmental analysis (Phoenix WinNonlin software, Certara, NJ, USA). PK parameters included maximum plasma concentration (C_max_), time to C_max_ (T_max_), area under the curve (AUC) from time zero to 24 h post dose (AUC_0–24_), AUC from time zero to the last measurable concentration (AUC_last_), AUC extrapolated to infinity (AUC_inf_), and terminal half-life (t_1/2_) where appropriate. Accumulation ratios (R_acc_) based on AUC_0–24_ were also calculated as the ratio of Day 14/Day 1 in Study 1.

### Statistical assessments and sample size

The dose-escalation within Study 1 followed a standard “3 + 3” patient enrolment scheme followed by an accelerated titration design, with safety and tolerability the primary objective. Therefore, no formal sample size calculation was made. No formal sample size calculations were made for Study 2, and a target of 6 volunteers was chosen which is consistent with other studies of this type. For Study 3, the sample size was calculated using a power of at least 90% and a 1-sided type 1 error of 5%. A true ratio between 95% to 105% was assumed, and an intra-subject coefficient of variation of approximately 28% was used. The power was defined as the probability of having a 90% confidence interval to a test/reference ratio for entrectinib C_max_ and AUC parameters within the acceptance criteria of 80% to 125%. In each part a total of 48 volunteers were to be dosed to allow for up to 4 volunteers overall as possible dropouts. Volunteers were allowed to participate in both Parts A and B of Study 3.

Exploratory statistical assessments of dose proportionality were conducted in Study 1 using the power model: Y = α • (dose)^β^, where Y is the PK parameter, and α and β are the intercept and slope, respectively [10]. After log-transformation, a mixed-effects statistical model was used to estimate α, β, and their 90% confidence intervals. Dose proportionality was assessed for the F1 formulation across the 100 to 400 mg/m^2^ dose range and using absolute doses (200 to 800 mg).

Statistical comparison of log-transformed C_max_, AUC_last_ and AUC_inf_ was conducted in Study 3 for assessment of bioequivalence and food effect. Results for each comparison were presented as ratios of geometric least squares mean and 90% confidence intervals. Bioequivalence, or lack of food effect was assumed if the 90% confidence intervals fell within the range 80% to 125%.

## Results

### Subject disposition and demographics

At the time of the data cut for Study 1 (31 May 2018), a total of 76 patients had been enrolled and treated, of whom 75 patients had evaluable PK data. Demographic characteristics were similar across the dose groups. Overall mean age was 54.3 (±14.99) years, the majority of patients were white (68.4%) or Asian (19.7%), and a similar number of male (47.4%) and female (52.6%) patients were enrolled.

A total of 7 male volunteers were enrolled and treated in Study 2, of whom 6 completed the study and were included in the ADME evaluations. The volunteers had a mean age of 28.7 (±6.58) years, a mean BMI of 27.8 (±3.27) kg/m^2^ and the majority were white (71%).

There was a total of 48 male volunteers in each of Parts 1 and 2 of Study 3, of which 13 volunteers participated in both parts. All volunteers completed Part 1, and 45 volunteers completed Part 2. The volunteers were predominantly white (88% in Parts 1 and 2). Mean age was 37.8 (±10.3) years and 39.1 (±9.21) years in Parts 1 and 2, respectively, and mean BMI was 26.7 (±3.36) mg/m^2^ and 26.9 (±2.90) mg/m^2^ in Parts 1 and 2, respectively.

### Pharmacokinetic results

#### Dose escalation study in adult patients with solid tumors (study 1)

**Fig. 1:**
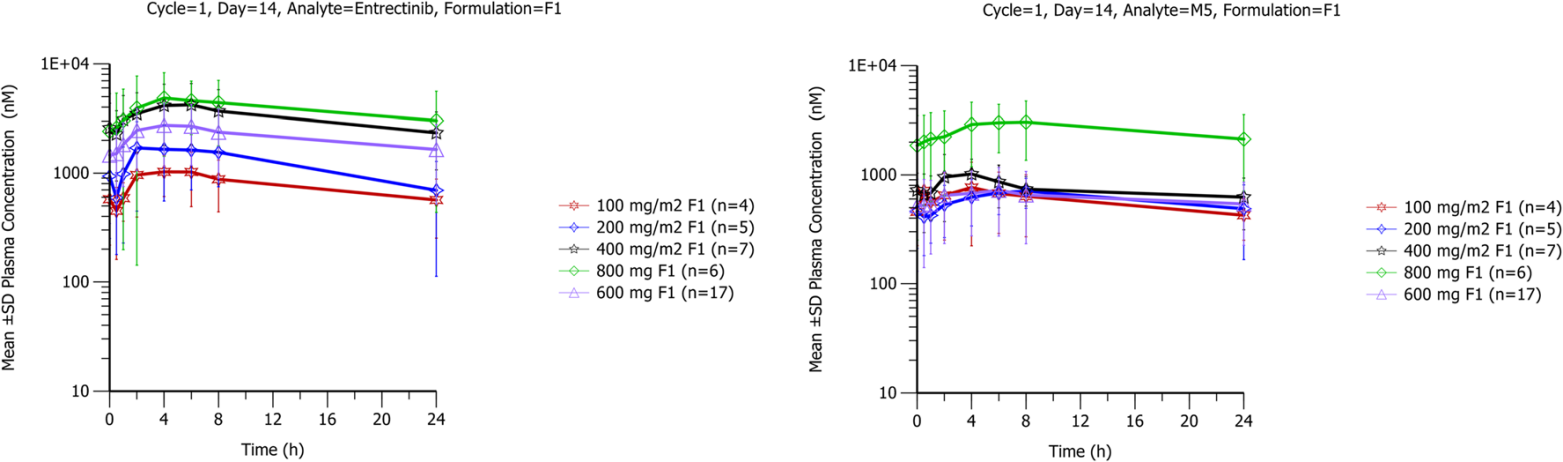
Mean (standard deviation) entrectinib and M5 plasma concentration-time profiles at steady-state in patients with advanced cancers after multiple doses of entrectinib (F1 formulation)

After single and multiple dosing of entrectinib in the fed state, plasma concentrations of entrectinib and M5 increased after dosing, with median T_max_ of 4 to 6 h (Table 1, Fig. 1). T_max_ was similar at 600 mg for both formulations (F1 and F2A). Entrectinib and M5 exposures (C_max_ and AUC parameters) increased with dose across the dose range tested. The dosing interval did not allow for determination of the terminal half-life via noncompartmental methods.

**Table 1:** Summary of entrectinib and M5 PK parameters after single and multiple doses of entrectinib in the fed state in patients with solid tumors

Entrectinib Dose	T_max_ (h)	Entrectinib			M5			
		C_max_ (μM)	AUC_0–24_ (μM•h)	Racc_(AUC0–24)_	C_max_ (μM)	AUC_0–24_ (μM•h)	M5/Entrectinib AUC ratio	Racc_(AUC0–24)_
Cycle 1, Day 1								
100 mg/m^2^; F1 (n = 5)	6.00 (4.00, 8.00)	0.506 (53)	7.17 (34)	N/A	NR^a^	NR^a^	NR^a^	N/A
200 mg/m^2^; F1 (*n* = 5)	6.00 (4.00, 8.00)	1.34 (47)	19.7 (42)	N/A	0.441 (55)	6.56 (61)	0.333 (75)	N/A
400 mg/m^2^; F1 (*n* = 10)	4.00 (2.00, 8.00)	2.52 (45)	38.0 (58)^b^	N/A	0.930 (76)^c^	17.8 (81)^d^	0.463 (101)^d^	N/A
800 mg; F1 (*n* = 9)	4.00 (4.00, 8.00)	3.41 (53)	49.6 (50)	N/A	1.41 (85)	23.0 (85)	0.463 (88)	N/A
600 mg; F1 (*n* = 22)	4.00 (1.00, 8.00)	1.87 (42)	22.3 (52)^e^	N/A	0.461 (95)	6.67 (91)^f^	0.276 (80)^f^	N/A
600 mg; F2A (*n* = 18)	4.00 (2.00, 8.00)	2.25 (58)	31.8 (48)^g^	N/A	0.622 (79)	10.2 (82)^g^	0.322 (49)^g^	N/A
Cycle 1, Day 14								
100 mg/m^2^; F1 (*n* = 4)	2.00 (2.00, 6.00)	1.04 (50)	16.8 (66)	2.08 (44)	0.680 (NC)^a^	12.6 (NC)^a^	0.549 (NC)^a^	NR^a^
200 mg/m^2^; F1 (*n* = 5)	6.00 (2.00, 8.00)	1.53 (80)	22.5 (97)^h^	1.15 (78)^h^	0.713 (43)	12.8 (60)^h^	0.569 (45)^h^	1.80 (26)^h^
400 mg/m^2^; F1 (*n* = 7)	4.00 (2.00, 6.00)	4.03 (60)	68.5 (65)	1.58 (24)^d^	0.892 (37)^i^	16.4 (37)^i^	0.273 (39)^i^	1.46 (79)^i^
800 mg; F1 (*n* = 6)	6.00 (2.00, 8.00)	4.72 (53)	77.3 (73)^d^	1.57 (23)^d^	2.91 (65)	49.6 (62)^d^	0.642 (62)^d^	2.59 (26)^d^
600 mg; F1 (*n* = 17)	4.00 (2.00, 8.00)	2.74 (58)	43.9 (64)^j^	2.11 (35)^j^	0.634 (76)	11.6 (76)^g^	0.265 (66)^j^	2.02 (77)^k^
600 mg; F2A (*n* = 12)	4.00 (2.00, 6.00)	3.13 (80)	48.0 (77)^l^	1.55 (49)^b^	1.25 (90)	24.0 (97)^l^	0.499 (142)^l^	2.84 (93)^b^

Values are geometric mean (geometric CV%), except T_max_ which is median (min, max)

F1 = early research formulation, F2A = research formulation used in pivotal studies

Dosing was conducted under fed conditions

^a^Not reported as less than 50% of patients had data; ^b^ *N* = 8; ^c^ N = 7; ^d^ N = 5; ^e^ *N* = 19; ^f^ N = 17; ^g^ *N* = 16; ^h^ N = 4; ^i^ N = 6; ^j^ *N* = 15; ^k^ *N* = 13; ^l^ N = 9

Steady state for entrectinib and M5 was achieved by Day 14. Geometric mean accumulation ratios based on AUC_0–24_ on Day 14 vs Day 1 ranged from 1.15 to 2.11 for entrectinib and 1.46 to 2.84 for M5, across the dose range of 200 mg/m^2^ to 800 mg.

Plasma concentrations of M5 were lower than entrectinib, with geometric mean M5 to entrectinib AUC_0–24_ ratios on Day 14 ranging from 0.27 to 0.64. The metabolite-to-parent ratios were broadly similar following both single and multiple dosing. Variability in entrectinib and M5 exposure parameters (C_max_ and AUC_0–24_) was high with coefficients of variation (CV%) being up to 97% on Days 1 and 14.

Dose proportionality assessments for entrectinib were conducted as described previously and the slope estimate was close to 1. At steady state, however, the 90% confidence intervals were wide due to the high variability, therefore, dose-proportionality was not formally demonstrated.

#### ADME study in healthy volunteers (study 2)

**Fig. 2:**
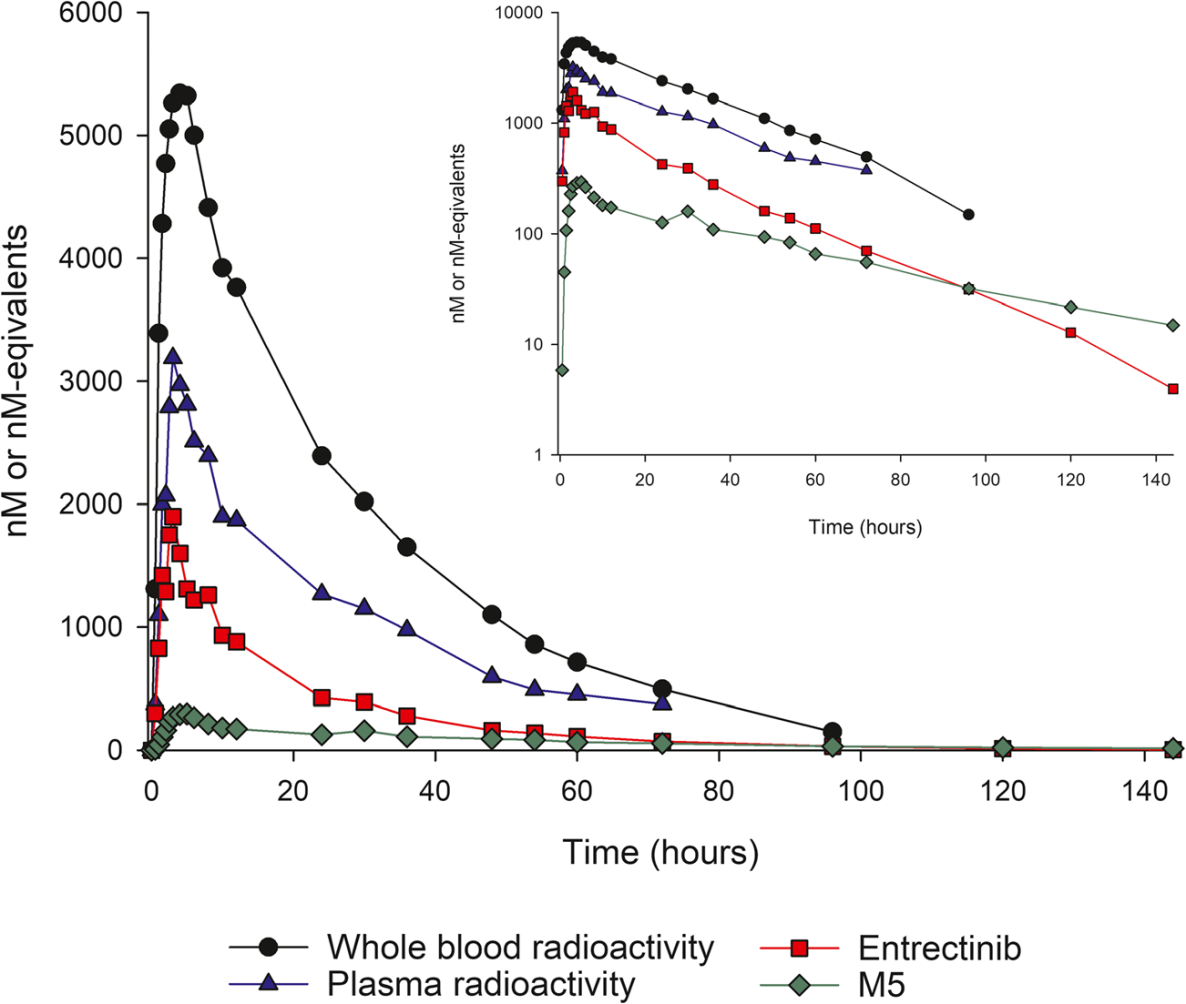
Median concentration-time profiles for radioactivity, entrectinib and M5 after a single dose of 600 mg [^14^C]-entrectinib in healthy volunteers

Following a single dose of oral [^14^C]entrectinib, radioactivity was readily absorbed with detectable radioactivity at 30 min postdose and median T_max_ occurring at 3–4 h in blood and plasma (Table 2, Fig. 2). Thereafter, blood and plasma concentrations declined in an approximately biphasic manner, with mean t_1/2_ of approximately 19 h.

**Table 2:** Summary of PK parameters for radioactivity, entrectinib and M5 after single dose [^14^C]entrectinib in healthy volunteers

Analyte	N	T_max_ (h)	C_max_ (μM)	AUC_0–24_ (μM•h)	AUC_inf_ (μM•h)	t_1/2_ (h)
Blood radioactivity	6	4.0 (2.5, 5.0)	5.38 (27)	90.3 (31)	180 (40)	22.5 (15)
Plasma radioactivity	6	3.0 (3.0, 8.0)	2.99 (37)	47.3 (46)	104 (54)	24.3 (16)
Entrectinib	6	3.0 (3.0, 4.0)	1.81 (38)	22.6 (42)	36.6 (47)	18.5 (17)
M5	6	4.0 (3.0, 6.0)	0.379 (67)	4.83 (67)	13.5 (61)	43.9 (16)

Values are mean (CV%) except for T_max_ which is median (min, max)

Dosing was conducted under fasted conditions

Mean blood-to-plasma ratios for C_max_ and AUC_inf_ were close to 2 and mean entrectinib-to-radioactivity ratios for C_max_ and AUC_inf_ in plasma were approximately 0.6 and 0.4, respectively.

Metabolic profiling of plasma samples showed that unchanged entrectinib was the most abundant drug-related circulating material representing 69% of total radioactivity in the 24-h period after a single dose of entrectinib. The metabolites M5 and M11 contributed 12% and 19% of total circulating radioactivity, respectively. Only one additional minor metabolite (M3; 1.3%) could be identified in plasma within the 24-h period. M5 was formed by N-demethylation of entrectinib, and had a longer half-life than entrectinib. M11 was a quaternary glucuronide conjugate formed by direct N-glucuronidation of entrectinib. Both M5 and M11 are considered to be major circulating metabolites of entrectinib in humans.

**Fig. 3:**
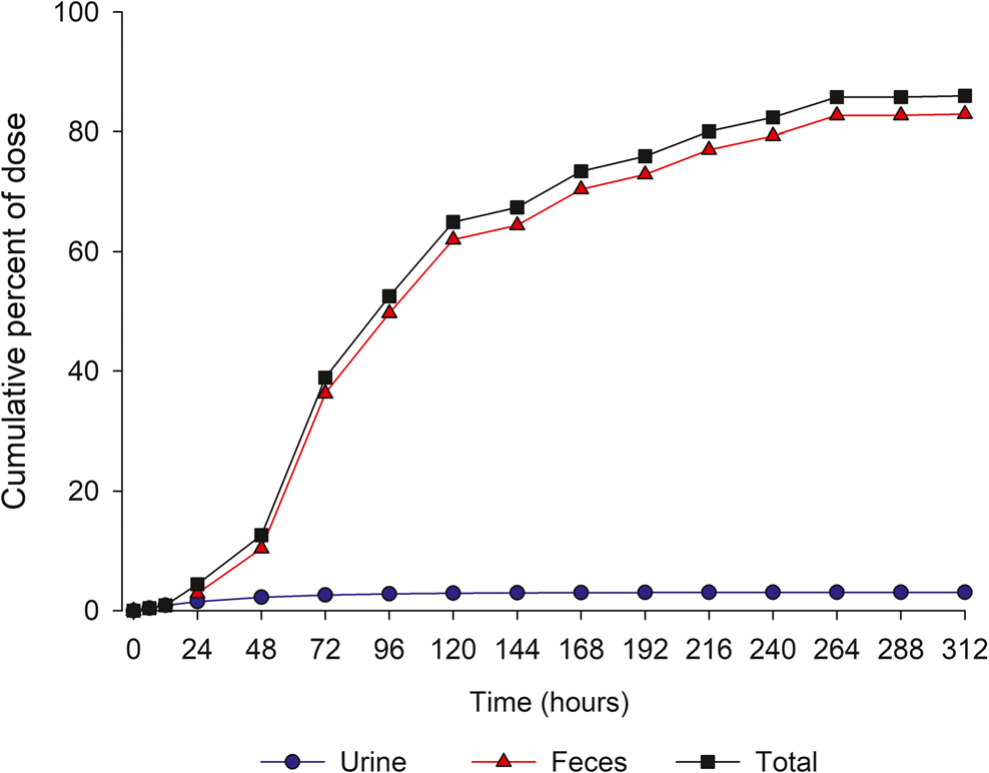
Cumulative mean recovery of radioactivity in urine and feces after a single dose of 600 mg [^14^C]-entrectinib in healthy volunteers

A total of 86% (range 72% to 91%) of radioactivity was recovered in urine and feces over the 312 h collection period. The majority of radioactivity (83%) was excreted in feces and approximately 3% of the dose was excreted in urine with less than 1% of the dose excreted in urine as unchanged entrectinib (Fig. 3).

In feces, entrectinib and M5 were the most abundant drug-related entities (36% and 22% of dose, respectively), followed by M1 (14%) and M2 (9%); M3, M4 and M11 were seen as minor components, collectively contributing less than 2% of the dose (Supplemental Table 1). M1 is the product of either mono oxidation of M5 or demethylation of M2. M2 is an oxidation product of entrectinib.

**Fig. 4:**
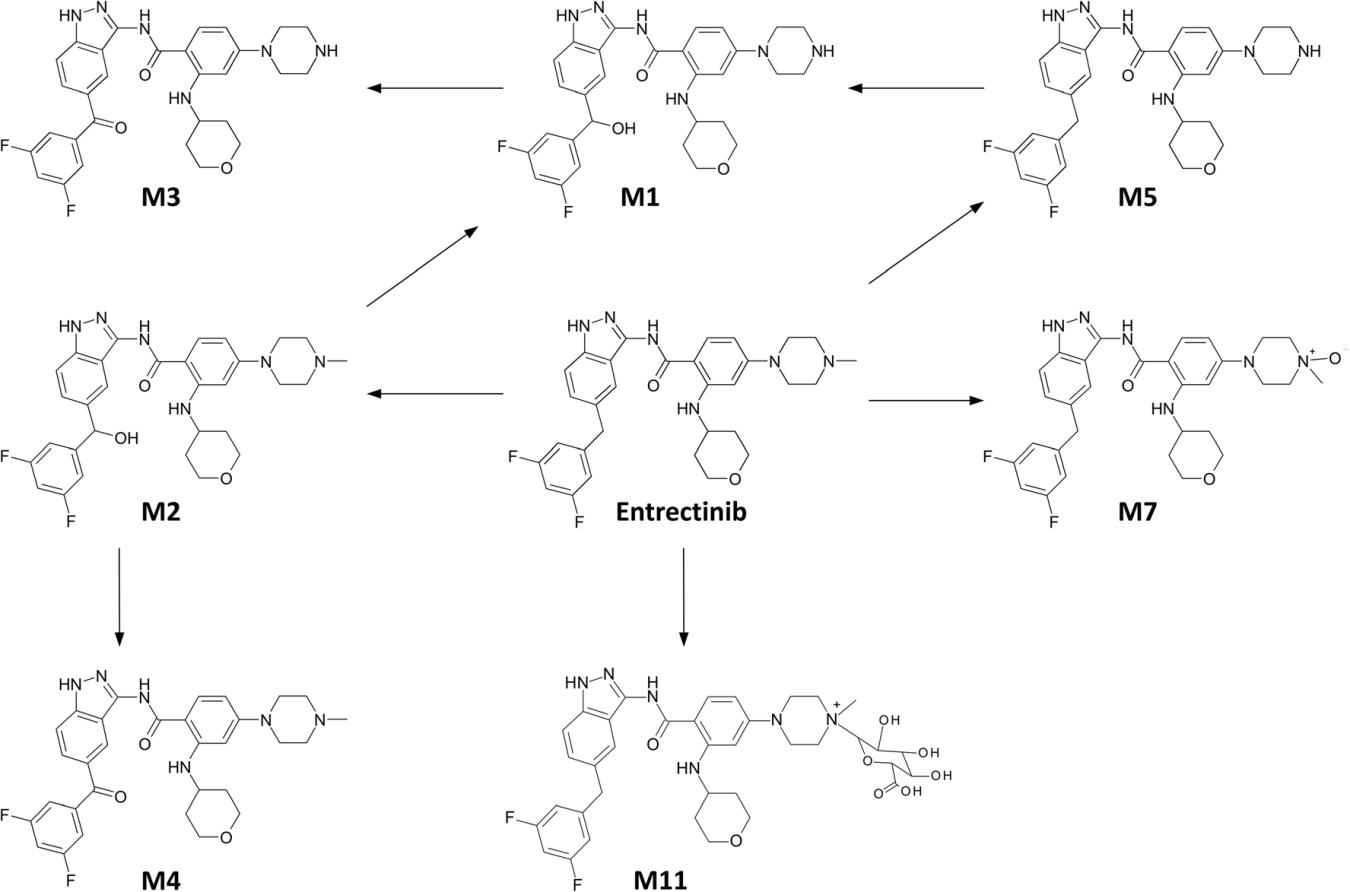
Proposed metabolic pathway for entrectinib in humans

The proposed metabolic pathway for entrectinib is presented in Fig. 4.

#### Bioequivalence study in healthy volunteers (study 3)

**Fig. 5:**
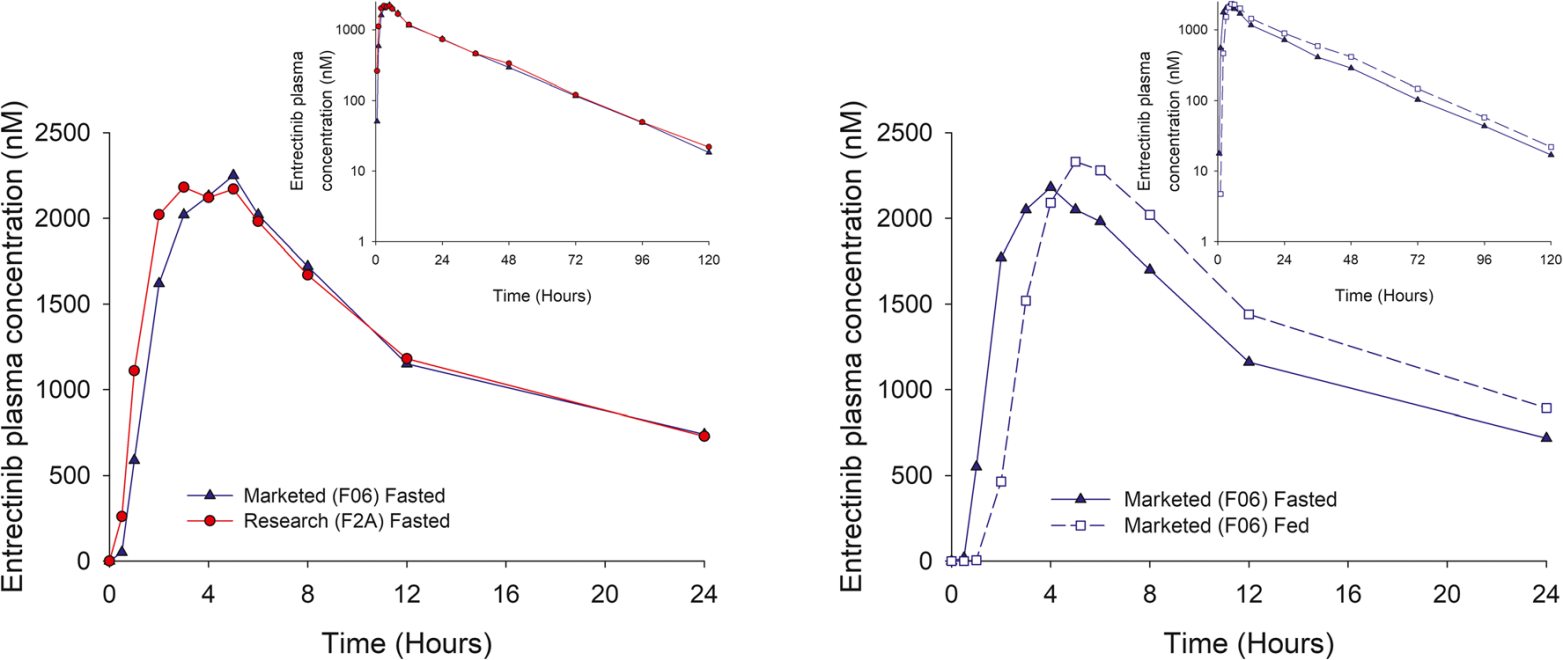
Median entrectinib plasma concentration-time profiles of 600 mg entrectinib administered as marketed and research formulations in the fasted state (left panel) and the effect of food on the marketed formulation (right panel)

In Part A, entrectinib plasma concentrations were similar following administration of the research (F2A) and marketed (F06) capsules in the fasted state (Table 3, Fig. 5 [left panel]). Median T_max_ was 3 and 4 h for the research and marketed formulations, respectively. Plasma concentrations declined with a mean half-life of ~19 h. Variability in entrectinib exposure was moderate to high with CV% for C_max_ and AUC parameters being 25 to 67%. M5 contributed approximately 30% of parent exposure, and had a mean elimination half-life of 36–38 h. Statistical assessment showed that the two formulations were bioequivalent with 90% confidence intervals for C_max_ and AUC parameter geometric mean ratios within the 80–125% range (Table 4).

**Table 3:** Summary of entrectinib and M5 PK parameters after single doses of 600 mg entrectinib administered as the research and marketed formulations in healthy volunteers

Analyte	Part	N	Treatment	T_max_ (h)	C_max_ (μM)	AUC_0–24_ (μM•h)	AUC_last_ (μM•h)	AUC_inf_ (μM•h)	t_1/2_ (h)
Entrectinib	1	48	Marketed (Fasted)	4.00 (2.00, 6.00)	2.18 (32)	29.0 (34)	47.6 (40)	48.3 (40)	19.2 (21)
		48	Research (Fasted)	3.00 (2.00, 5.00)	31.7 (26)	31.7 (26)	52.1 (34)	52.8 (34)	19.1 (25)
	2	46	Marketed (Fed)	5.00 (3.00, 8.00)	2.38 (25)	32.1 (25)	57.2 (30)	57.9 (31)	18.8 (23)
		47	Marketed (Fasted)	4.00 (2.00, 6.00)	2.25 (33)	29.8 (34)	50.5 (67)	51.2 (37)	18.2 (18)
M5	1	48	Marketed (Fasted)	5.00 (3.00, 6.00)	0.451 (41)	5.22 (42)	12.4 (38)	13.9 (38)	37.8 (14)
		48	Research (Fasted)	4.50 (3.00, 6.00)	0.465 (38)	5.49 (36)	13.0 (32)	14.4 (32)	36.3 (15)
	2	46	Marketed (Fed)	5.00 (5.00, 8.00)	0.403 (34)	5.09 (34)	13.9 (34)	15.7 (35)	38.8 (29)
		47	Research (Fasted)	5.00 (4.00, 6.00)	0.442 (46)	5.04 (46)	12.4 (42)	14.0 (41)	36.3 (16)

Values are geometric mean (geometric CV%) except for T_max_ which is median (min, max), and t_1/2_ which are arithmetic mean (CV%)

**Table 4:** Summary statistics for assessment of bioequivalence and food effect for entrectinib in healthy volunteers

Comparison	PK Parameter	Geometric LS mean ratio	90% Confidence Intervals
Marketed vs Research (Fasted)	C_max_	93.3	88.3, 98.6
	AUC_last_	91.4	85.3, 97.9
	AUC_inf_	91.4	85.4, 97.9
Marketed (Fed) vs Marketed (Fasted)	C_max_	106	98.9, 115
	AUC_last_	115	107, 123
	AUC_inf_	115	107, 124

In Part B, entrectinib plasma concentrations were similar following administration of the marketed capsules in the fasted and fed states (Fig. 5 [right panel]). Median T_max_ was 4 h in the fasted state and 5 h in the fed state. Statistical assessment showed that there was no clinically relevant effect of food (a high-fat, high calorie meal) on entrectinib exposure when administered as the marketed formulation, with 90% confidence intervals for the C_max_ and AUC parameter geometric mean ratios within the 80–125% range (Table 4).

## Discussion

Entrectinib has been investigated in adults over a wide dose range, initially at dose levels based on body surface area (100 to 400 mg/m^2^) and subsequently at flat doses of 600 and 800 mg. The recommended adult dose is 600 mg once daily. Entrectinib exposure increased with increasing dose and, independent of dose, achieved peak concentrations within approximately 3 to 6 h after oral dosing in adults. Entrectinib is the major circulating entity, with M5 (active metabolite) being a major circulating metabolite with metabolite/parent ratios in plasma of 0.27 to 0.64. Steady-state was achieved for both parent and M5 major active metabolite within 14 days of dosing. The half-life of M5 was approximately twice that of entrectinib, however, despite the difference, the observed accumulation ratios were similar for both compounds, suggesting that the terminal phase does not contribute to M5 exposure to a significant extent.

It was not possible to conclude dose-proportionality in entrectinib PK in Study 1. This is thought to be partly due to the high variability observed in entrectinib PK with the original F1 formulation. It should also be noted that the dose-escalation design, and small sample sizes involved in Study 1 were not ideal for this assessment. A posthoc analysis has subsequently been performed on pooled entrectinib exposure data from 10 single dose studies in healthy volunteers which confirmed dose proportionality across the 100 to 800 mg dose range (Roche data on file).

Oral dosing with [^14^C]entrectinib confirmed that entrectinib was well absorbed when administered with an acidulant. Absolute bioavailability was estimated to be >50%. This is based on physiologically-based PK modeling (Roche data on file) and is consistent with the ADME data that suggests >53% of entrectinib is absorbed assuming that all fecal metabolites originated from systemic metabolism of entrectinib.

Mean blood-to-plasma ratios for C_max_ and AUC_inf_ were close to 2, indicating most radioactivity was associated with red blood cells. The mean entrectinib-to-radioactivity in plasma ratios for C_max_ and AUC_inf_ (0.6 and 0.4, respectively) indicate that the majority of circulating radioactivity at C_max_ was entrectinib, but overall metabolite exposures (AUC) were greater than the parent compound. Metabolite profiling indicated that entrectinib was the most abundant circulating entity, with M5 and M11 being major circulating metabolites. M5 is formed by oxidation and is an active metabolite (in vitro data), therefore bioanalytical assessment was mandatory to demonstrate coverage in animal species according to the MIST guidance. [11] The case of M11 is different since it is not active and an N-glucuronide conjugate does not require bioanalytical quantitation in humans or in animal species used for toxicity testing of entrectinib.

Entrectinib is cleared mainly through metabolism and both entrectinib and its metabolites are eliminated mainly in feces. Minimal renal excretion was observed with only 3% of the administered radioactive dose being excreted in urine.

Under controlled dosing conditions, entrectinib and M5 PK were similar in patients with cancer and healthy volunteers; entrectinib AUC_0–24_ was approximately 22 to 32 μM•h in Study 1 (patients with cancer), compared with approximately 23 to 32 μM•h in Studies 2 and 3 (healthy volunteers).

Entrectinib is a lipophilic base with high aqueous solubility at low pH but shows a pronounced decrease in solubility as pH increases [8]. In order to improve solubility and reduce the high variability observed with the early formulation (F1) in Study 1, an acidulant was added to the research formulation F2A (and the marketed formulation F06). Comparison of C_max_ and AUC_0–24_ values with 600 mg entrectinib dosing in Study 1, suggests that both entrectinib and M5 exposure were higher following administration as the F2A research formulation. Although variability was not notably improved with the F2A formulation in Study 1, the studies in healthy volunteers suggested that variability with the F2A and F06 formulations was moderate. The F2A formulation was subsequently used in the pivotal studies with entrectinib which confirmed its anti-tumor activity.

The use of the acidulant in the entrectinib formulations was also designed to limit the potential for interactions with food (and modulators of gastric pH such as proton pump inhibitors). This was confirmed in Study 3, where dosing with food was shown to have no clinically relevant effect on entrectinib PK.

Bioequivalence was demonstrated under fasted conditions between the marketed capsule formulation (F06) and the research capsule formulation (F2A) used in the pivotal studies.

Overall, these studies enable a thorough understanding of the pharmacokinetics and disposition of entrectinib, indicating that entrectinib is well absorbed, with linear PK, suitable for once daily dosing, and can be taken with or without food. In addition, the clinical implications of entrectinib disposition support the notion that renal impairment is unlikely to significantly impact exposure to entrectinib, whereas hepatic impairment may potentially alter exposure to the parent drug and/or metabolite(s).

## Supplementary Information

ESM 1 (DOCX 12 kb)
