# Supplementary material for: Characterization of the pharmacokinetics of entrectinib and its active M5 metabolite in healthy volunteers and patients with solid tumors
Source: Invest New Drugs. 2021 Jan 18;39(3):803–11. doi: 10.1007/s10637-020-01047-5 (PMC8068699; doi:10.1007/s10637-020-01047-5)
Supplement: Supplementary file 1 — (DOCX 12 kb) [file 10637_2020_1047_MOESM1_ESM.docx]

SUPPLEMENTAL MATERIAL

Supplemental Table 1: Metabolic profile of entrectinib in human urine and feces

| **Matrix** | **% of Dose** | | | | | | | | | |
| --- | --- | --- | --- | --- | --- | --- | --- | --- | --- | --- |
|  | **M1** | **M2** | **M3** | **M4** | **M5** | **M7** | **M8** | **M11** | **Entrectinib** | **Total** |
| Urine | 0.482 | 0.782 | 0.0312 | ND | 0.171 | 0.279 | 0.488 | 0.165 | 0.663 | 2.40 |
| Feces | 14.2 | 9.45 | 1.22 | 0.185 | 22.1 | ND | ND | 0.0583 | 35.7 | 86.0 |
| Sum | 14.7 | 10.2 | 1.25 | 0.185 | 22.2 | 0.279 | 0.488 | 0.224 | 36.4 | 88.4 |

ND = not detected
